# Effects of occupational heat exposure on female brick workers in West Bengal, India

**DOI:** 10.3402/gha.v7.21923

**Published:** 2014-02-03

**Authors:** Moumita Sett, Subhashis Sahu

**Affiliations:** 1 Ergonomics and Occupational Physiology Laboratory, Department of Physiology, University of Kalyani, Kalyani, West Bengal; 2 Department of Physiology, Naiminath Homoeopathic Medical College, Hospital & Research Centre, Agra, India

**Keywords:** cardiac strain, heat exposure, workload, productivity, female brickfield workers

## Abstract

**Background:**

Manual brick-manufacturing units in India engage a large number of female workers on a daily-wage basis for a period of 8 months per year. There are two groups of female workers in the brickfields: the brick molders and the brick carriers. These brickfields are mostly unorganized, and the workers are exposed to extreme conditions such as very high seasonal heat. The present trend of increasing temperatures, as a result of global warming and climate change, will put an additional burden on them.

**Objective:**

This study aims to evaluate the effect of workplace heat exposure on the well-being, physiological load, and productivity of female brickfield workers in India.

**Design:**

A questionnaire study (*n*=120), environmental temperature, and weekly work productivity analyses were evaluated for 8 months in the brickfields. Cardiac strain and walking speed (subset, *n*=40) were also studied and compared in hotter and colder days amongst the female brickfield workers.

**Results:**

The subjects experience summer for about 5 months with additional heat stress radiating from the brick kiln. The weekly productivity data show a linear decline in productivity with increased maximum air temperature above 34.9°C. The cardiac parameters (peak heart rate (HRp), net cardiac cost (NCC), relative cardiac cost (RCC), and recovery heart rates) were significantly higher on hotter days (Wet Bulb Globe Temperature (WBGT_out_) index: 26.9°C to 30.74°C) than on cooler days (WBGT_out_ index: 16.12°C to 19.37°C) for the brick molders; however, this is not the case for the brick carriers. As the brick carriers adapt to hotter days by decreasing their walking speed, their productivity decreases.

**Conclusion:**

We conclude that high heat exposure in brickfields during summer caused physiological strain in both categories of female brickfield workers. A coping strategy employed by the brick carriers was to reduce their walking speed and thus lose part of their earnings. The lost productivity for every degree rise in temperature is about 2% in the brickfields. This reduction will be exacerbated by climate change and may undermine the quality of life of female brickfield workers.

Brick-making work has dominated construction in India since antiquity. The lure of steady work draws migrant labor families from the villages of the same or other states that come to these brick-making units temporarily for a period of about 8 months per year. Also, some migrant workers come to the brickfields who can no longer sustain themselves by farming (1). During the remaining months of the year (i.e. from June to September), it is monsoon season in West Bengal, India, with intermittent or frequent showers of rainfall. So, during monsoons these brick-making units remain closed as sun drying the bricks is impossible in these months.

Manual brick-making units employ millions of female workers with a few male supporting staff (2). There are two groups of female workers: the *brick molders*, who only collect mud to mold bricks and lay them on the field for sun drying; and the *brick carriers*, who carry sundried and baked bricks to and from the kiln. The work of each group is not interchangeable (3).

The female workers not only are continuously exposed to the sun but also are exposed to an extra amount of heat that is exchanged by radiation and convection both inside and outside the brick kiln (4). Additionally, female workers have specific stress-related disorders resulting from job discrimination and a double burden of work (in the workplace and home) (5). For example, after working in the field, their male counterparts go back to take rest, but the female workers have to manage their children and cook food for their family as well. Again, the males are given more food (or milk, etc.) than the female counterparts. Thus, the males altogether have a better ability to work and give their full efficient output than the females.

It is a well-known physiological fact that human performance is limited by excessive environmental heat exposure (6, 7). Based on the international standard for maximum recommended heat exposure during continuous work of high physical intensity (8), it was concluded that physical labor will become more difficult for millions of working people as climate change progresses (9). Climate change and increased heat have several effects on the human physiological system (9).

Various factors have already been reported to influence the cardiovascular system (10, 11). Among them, dehydration and heat have been recognized as two factors that can influence the heart rate (12). Numerous studies have been carried out all over the world in the field of industrial heat stress that prove that heat stress leads to the deterioration of performance, efficiency, production, and thus quality of work (13–15). Moreover, gender differences in thermoregulation become more apparent with the greater thermal loads (16). Lower aerobic capacities for women increase the relative workload of a given task, and smaller blood volumes in women result in higher heart rates (17).

Heat and humidity not only act on human physiology, but also reduce work productivity, condensing the world's economic productivity and particularly affecting the developing countries in the tropical climate zone (18, 19). Excessive heat exposure is a health risk for all age groups (20). Kjellstrom et al. (9) described the physiological mechanisms behind health and productivity effects. A study shows that the largest proportion of heat effects is not reported cases of hyperthermia, but mainly increases in cardiovascular and respiratory hospitalizations and deaths (21). Beyond the acute heat stress, more chronic effects on the heart and kidneys may develop after repeated excessive body heating or dehydration (7).

There are few studies on the relationship between heat exposure and worker health impacts and productivity (22–25) in different industrial settings, and there is a dearth of data regarding the cardiac strain on female brickfield workers due to various factors. Thus, in India there are still very little data regarding thermal stress and its relationship with changes in heart rate, and the data that are available are neither very recent nor related to female workers of the unorganized labor sectors. Thus, the objective of this study is to evaluate the effect of workplace heat exposure and workload on the subjects’ health and productivity.

## Methods

### Subject selection

The study was conducted in three brickfields of West Bengal for three consecutive summer and winter seasons. West Bengal is a state situated in the eastern zone of India. The three brickfield sites are present in the three districts of Nadia, Hooghly, and Howrah. Each field employed about 40 to 50 female workers in each session. But the physiological exploratory studies were conducted amongst 22 female brick carriers and 18 brick molders in different brick-making units. All persons gave their informed consent prior to their inclusion in this study. The experiments were done according to the ethical standards of the Departmental Research Committee of Kalyani University.

Thus, the study was conducted from October 2008 to May 2009 (first session), from October 2009 to May 2010 (second session), and then from October 2010 to May 2011 (third session). West Bengal, India, experiences winter from mid-November to mid-February, and the rest months of the working season are included within the summer months. Thus, each session included two seasons within it (i.e. a total of six seasons – three winter and three summer seasons).

### Questionnaire study

Preliminary data were collected from 120 female brickfield workers through interview and questionnaire methods (i.e. the Hothaps questionnaire) (26). As the majority of the subjects were illiterate, the questionnaire was conveyed to the subjects in their local languages (i.e. Bengali and Hindi). This study was carried out in the first session (i.e. once in each season) and on the same number of female subjects.

### Physical parameters

Weights of the subjects were measured in kilograms by a properly calibrated digital weighing machine, and heights were measured in centimeters, without footwear, and using a standardized anthropometric rod. The physical parameters were mostly measured during their holidays (i.e. Sunday) or sometimes during their lunch hours.

### Measurement of the environmental parameters

The main heat exposure parameter surrounding the brick kiln was air temperature (Ta, or dry bulb temperature) measured with an LCD Portable Digital Multi-Stem Thermometer with an external sensing probe (Model No. ST-9269, India) shielded from the sun and close to the workers. The temperature was recorded on Wednesday (i.e. the middle of every week) for 7 hours a day, in every month of each session at the same manual brick-manufacturing unit.

A globe thermometer was used to measure the Tg (globe temperature), and a mercury thermometer was used to measure the natural wet bulb temperature (Tpnwb). Thus, Wet Bulb Globe Temperature (WBGT) was calculated (via the WBGToutdoor index) by the standard formula of Parsons (6). The WBGT index was recorded hourly in days when cardiovascular parameter and walking speed were studied.

Weather station reports of Kolkata, the capital of West Bengal, were downloaded at intervals of every fortnight, but the record was on a daily basis (27).

### Productivity analyses

Tally counters calculated the number of bricks laid down on the field by the brick molders and the number of times that the brick carriers walked to and from the brick kiln, loaded with bricks. At one time, the carriers carried 8–10 bricks, and it varied from individual to individual. According to the individual's capacity of carrying, some of them carry either 8 bricks or 10 bricks. This is because they carry bricks in an arrangement that requires either 10 bricks or 8 bricks. Carrying fewer than eight bricks means losing a part of their earning, because they earn according to the number of bricks they carry in a day. Regarding the tally recording, the workers were not told about the study in order to avoid bias in the productivity of individual workers who worked according to their usual capacity. However, throughout the 8-month working period, their productivity was recorded on a weekly basis from the record register book (brick molders = 88 and brick carriers = 32; *N =*120) for three sessions, and it was calculated as productivity per person per week.

### Cardiovascular parameters

The following parameters were taken only in the third session after assessing the whole conditions in the first two sessions. The study was conducted on the same group of brick molders (*N*=18) and brick carriers (*N*=22) in two seasons.

#### Resting heart rate (HR_rest_)

The subjects were allowed to rest for a period of 30 min. Following the rest period, the heart rate monitor (Polar Accurex Plus, Polar electro Oy, S810i, Finland) was worn over and recorded for 5 min. The minimum value obtained during this period was considered as the resting value.

#### Pre-working, working, and recovery heart rate

The subjects were asked to perform their work for a time period of 30 min. No restriction was imposed on the speed of carrying or molding, and the subjects worked at their capacity. The subjects were allowed to take sufficient rest before starting the activity to determine the resting heart rate (HR_rest_) or, rather, the pre-working heart rate (WHR). During their working period (30 min), heart rate was continuously recorded every minute during this period, and the recovery heart rate was also recorded until they attended the resting heart rates. The heart rates were recorded many times with a heart rate monitor to get an exact mean and to reach a definite conclusion.

#### Peak heart rate (HRp)

This is the maximum heart rate recorded during the test.

#### Average working heart rate (AWHR)

Estimated from the value of the 4th to the 30th min of work (28).

#### Sum of recovery heart beats (SRHB)

This is a measure of work strain that was calculated by summing the heart rate values during the recovery period of 10 min (29).

#### Cardiac strain

Net cardiac cost (NCC) and relative cardiac cost (RCC) were considered as two derived indices of cardiac strain (30).

### Calculation of walking speed

A pedometer (Omron Step Counter, Model HJ-113, Japan) was hung around the waist of the female brick workers to record the number of steps that an individual took, and thus the distance as well as the speed were recorded while the subjects walked with or without a load in the brickfield. The study was conducted on 40 subjects twice in hotter and cooler days, along with the recording of WBGT.

### Statistical analysis

The mean and standard deviation of various physical and physiological parameters were calculated. Then, a student's *t*-test or one-way analysis of variance (ANOVA) was done to find the significant difference between the measured physiological parameters for the chosen level of significance (*p*<0.05) if it was not otherwise mentioned (31). The measurements in summer and winter were assumed independent, and, thus, a paired test was not used because the readings in the summer and in the winter months were samples that were not necessarily gathered from the same individual. The analyses are therefore on the mean of all the study participants, and so ANOVA was used to study differences.

## Results

### Questionnaire study

Results of the Hothaps questionnaire analyses have been summarized and tabulated in Table 1.

**Table 1:** Results of questionnaire analysis (N=120)

Questions	Responses (%)
Awareness of heat stress symptoms	71.67
Discomfort during hottest days	99.17
Exhaustion during hottest days	94.17
Work productivity loss during hot seasons	89.17
Forced to work on hot days due to poverty	90.00
Productivity affects income on hotter days	98.33

N.B.: The percentage does not add up to 100% due to multiple responses.

This preliminary data helped to further elaborate the study.

### Physical characteristics

The physical characteristics of the female subjects are tabulated in Table 2.

**Table 2:** Physical characteristics of the female subjects

Parameters	Brick carriers (*n*=22), Mean±*SD*	Brick molders (*n*=18), Mean±*SD*	ANOVA significance
Age (years)	21.3±1.3 (18–23)	26.5±3.4 (19–30)	*p*<0.05
Height (cm)	146.4±3.25 (140.3–152.6)	145.1±3.33 (140.56–149.8)	NS
Weight (kg)	36.32±4.11 (26.7–40.5)	35.9±3.56 (24.8–38.37)	NS
Experience (years)	4.3±0.6 (0.5–5.0)	8.1±1.7 (2.5–6.2)	*p*<0.01

Range is shown in the parentheses.

The age (*p*<0.05) and the experience (*p*<0.01) of the brick molders are significantly higher (Table 2) than those of the brick carriers, and it has been observed that they leave the job after a few years. The molders are mostly married women who work faster to carry out their household jobs as well. In contrast, the carriers are very young and unmarried, so they do not have as much of a burden of household chores.

The brick carriers are young in age (21.3±1.3 years old), and their mean experience in brick carrying is about 4 years, ranging from 1.0 to 5.0 years. However, although they have at least 1 year of work experience, they are still unaware of the safety rules and so they do not take up any preventive measures, as is also seen from the questionnaire study.

### Environmental parameters

**Fig. 1:**
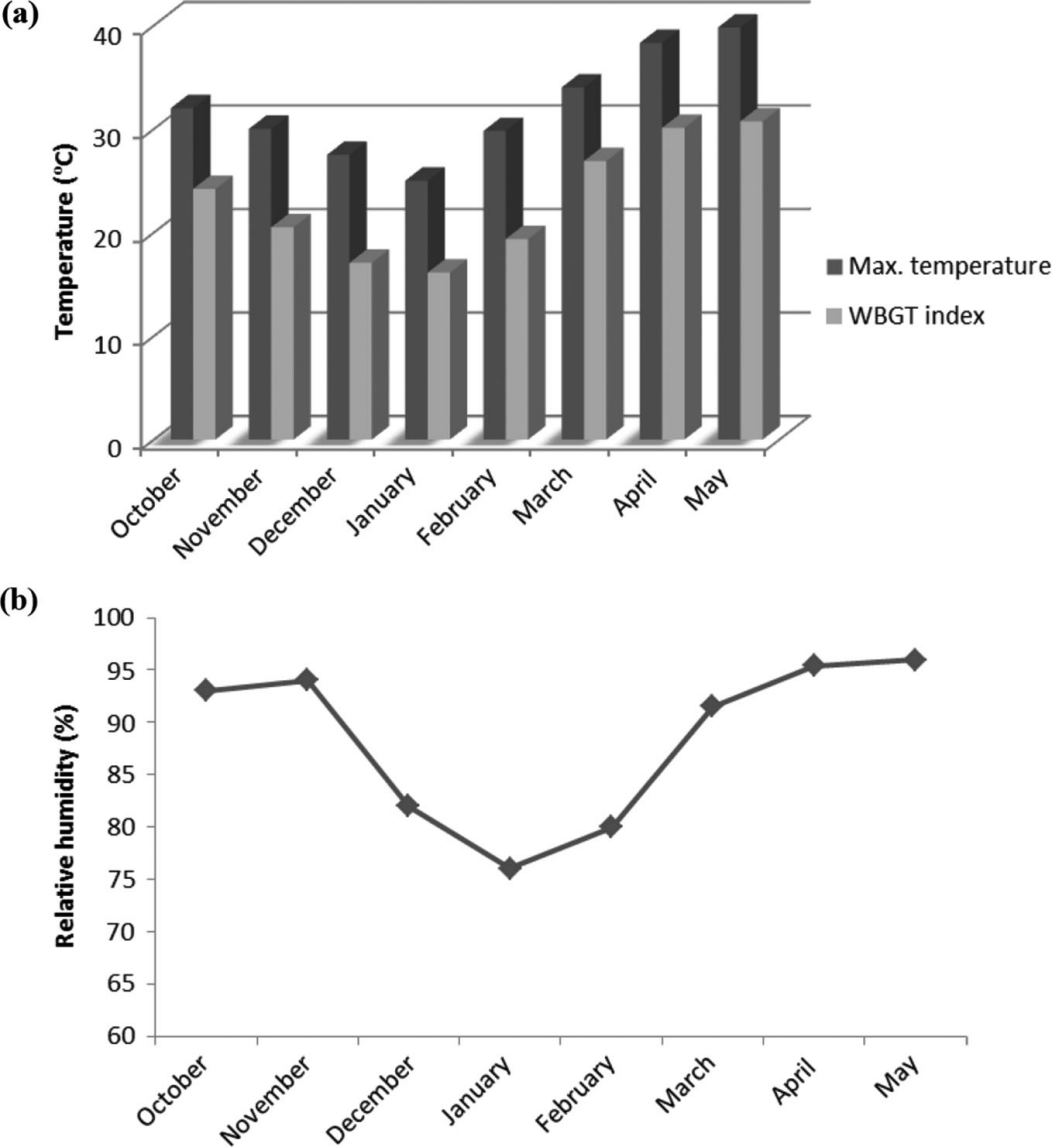
Thermal environmental variations (a, b) in the brick-manufacturing units.

Figure 1 (a) and (b) shows the average minimum and maximum temperatures and minimum and maximum relative humidity of the working months of the female workers in the manual brick-making units of the three sessions.

It is seen from the records of the two sessions and of the two seasons that the temperature reaches its minimum in January (11.2±1.7°C), followed by December (15.3±2.11°C), and its maximum is in the month of May (39.8±4.81°C). Even the relative humidity is minimum in December and January (39%), and the maximum is in the months of April and May (95%). From mid-November to mid-February, one experiences winter in West Bengal, India, and the rest of the months of the working season are summer months.

### Productivity analyses

**Fig. 2:**
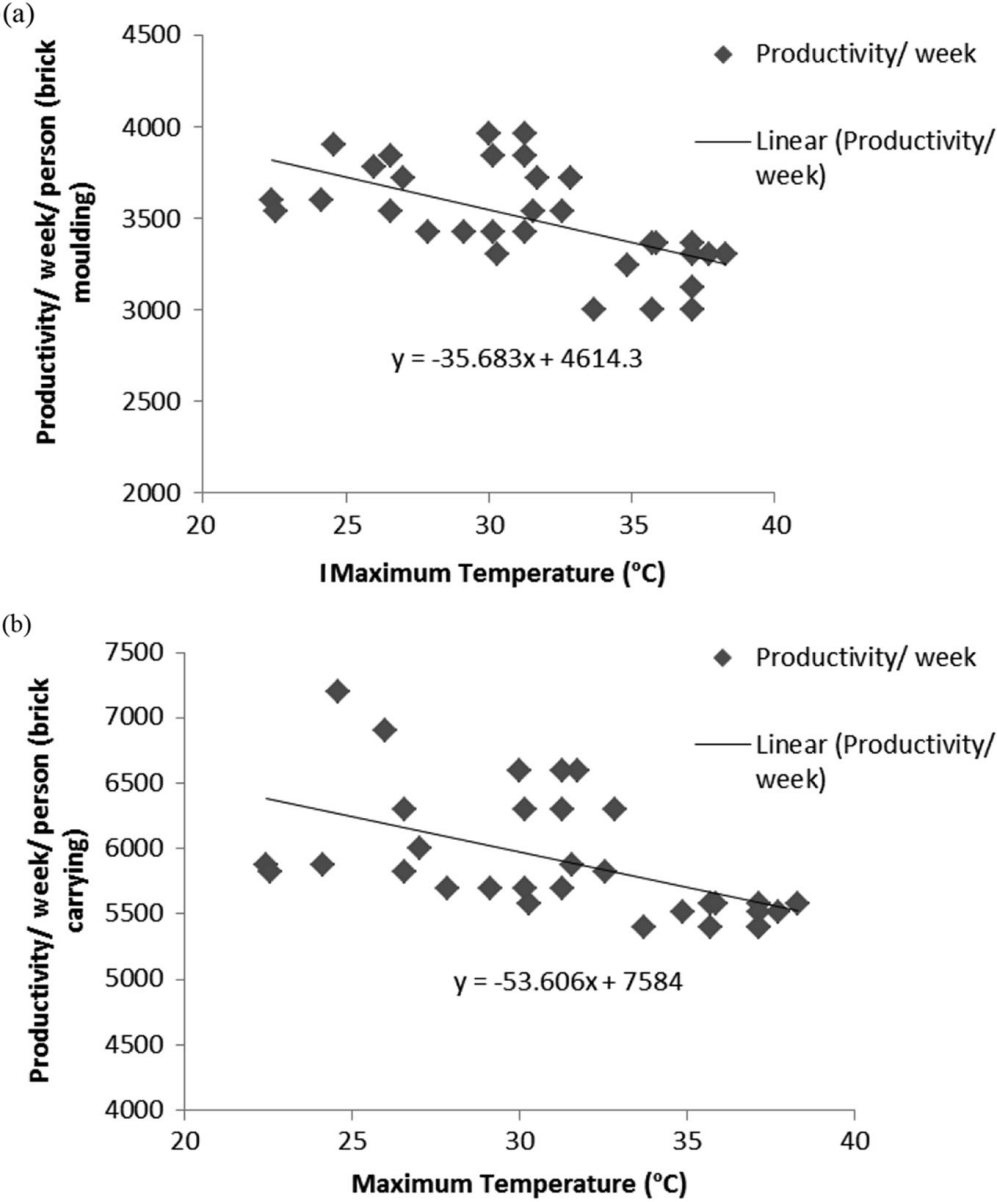
Productivity of the (a) brick molders (*n*=88) and the (b) brick carriers (*n*=32) with respect to temperature changes in the working months.

Figure 2 shows the relationship of the mean maximum temperatures and the mean weekly productivity per person for the brick molders (*n*=88) (Fig. 2a) as well as the brick carriers (*n*=32) (Fig. 2b).

**Fig. 3:**
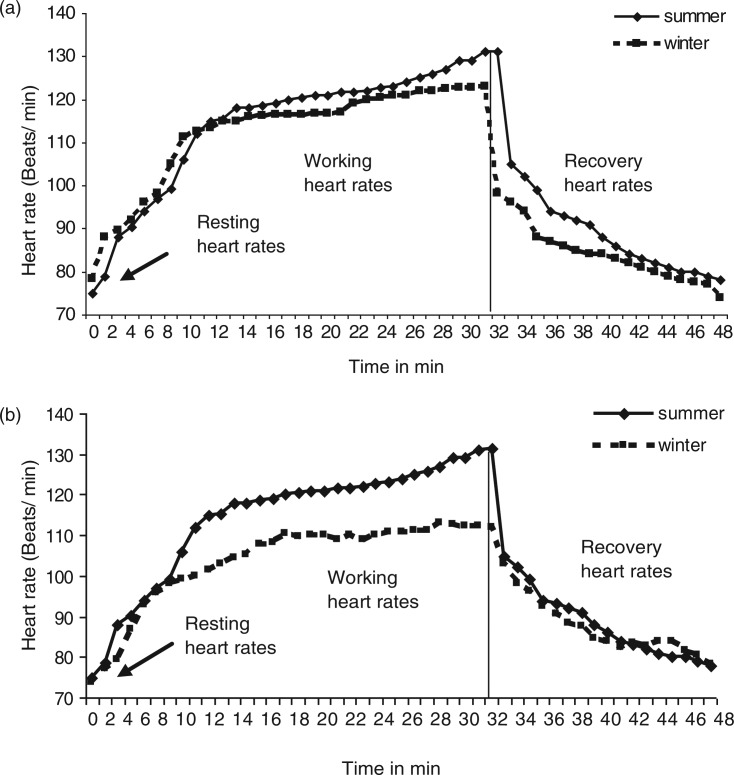
Variations of the working heart rates (beats/min) of the (a) brick carriers (*n*=22) and (b) the brick molders (*n*=18) in two seasons.

Brick molders’ productivity includes molding bricks and laying them on the field to dry, as measured per week, whereas productivity of the sun-dried and baked brick carriers includes carrying bricks to and from the brick kiln. In each session, it is seen that with increases in the environmental temperature (as collected from the weather station), the productivity of the brick molders as well as the brick carriers decreased proportionately, and they worked until they were fully exhausted. Only then did they take rest for 10–15 min in the shades of the trees, then resume work. Incidents of being exhausted naturally occur more in the summers than in the winter months.

### Cardiovascular parameters

Variations of the mean WHRs (beats/min) of the brick carriers (*n*=22) and brick molders (*n*=18) in two seasons have been graphically shown in Fig. 3. This study was performed after they resumed their work after lunch, in each season of the third session. During the cardiovascular parameter study period, the average WBGT_out_ index in the winter months ranged from 16.1°C to 19.4°C, whereas in the summer months it ranged from 26.9°C to 30.7°C.

The resting heart rates of the brick carriers (81.0±4.25 beats/min) and the brick molders (74.91±3.57 beats/min) in summer are significantly higher (*p*<0.01; *p*<0.05) than the heart rates of the same subjects measured in winter (76.6±3.7 and 72.33±2.19 beats/min, respectively).

Different cardiovascular parameters and their statistical analyses have been tabulated in Table 3.

**Table 3:** Cardiovascular parameters of the two groups of workers in two seasons

Groups	Parameters	Winter (WBGT_out_ index: 16.1°C to 19.4°C), Mean±*SD*	Summer (WBGT_out_ index: 26.9°C to 30.7°C), Mean±*SD*	ANOVA significance
Brick carriers	SRHR (beats)	1552.25±12.32	1552.97±16.88	NS
(*n*=22)	NCC (beats)	1257±122.69	1259.64±136.96	NS
	RCC (%)	81.40±3.16	83.58±5.08	NS
	HRp (beats/min)	123.55±5.11	124.93±10.82	NS
Brick molders	SRHR (beats)	798.21±100.80	995.85±16.83	*p*<0.0001
(*n*=18)	NCC (beats)	923.36±135.61	1091.64±116.53	*p*<0.001
	RCC (%)	75.77±5.99	80.54±3.03	NS
	HRp (beats/min)	115.25±9.04	121.85±5.13	*p*<0.05

In each of the cardiac strain parameters tabulated in Table 3, it is seen that the values are significantly higher in summer than in winter. Surprisingly, the cardiac cost of the brick carriers is almost similar both in summer and in winter, which shows that their workload is much greater compared to that of the female brick molders. ANOVA results showed that there was a significant difference in HRp (*p*<0.05), NCC (*p*<0.001), and SRHR (*p*<0.0001) values of the brick molders, which were sampled at two seasons of their work in the brickfield.

### Analyses of the walking speed

Table 4 shows the variations of speed (km/hour) in two seasons while walking with or without a load, and Table 5 shows the statistical significance between the walking speeds (km/hour) of the two groups of workers in the two seasons: winter (WBGT_out_ index: 16.1°C to 19.4°C) and summer (WBGT_out_ index: 26.9°C to 30.7°C) of the first session.

**Table 4:** Observed variations of speed (km/hour) in two seasons while walking with or without a load of bricks

	Summer (WBGT_out_: 26.9–30.7°C)		Winter (WBGT_out_: 26.9–30.7°C)	
Groups	With load	Without load	With load	Without load
Brick carriers (*n*=22)	2.0±0.21	3.8±0.15	3.3±0.1	4.8±0.3
Brick molders (*n*=18)	2.9±0.1	4.1±0.7	3.8±0.07	5.4±0.13

**Table 5:** Statistical significance between the observed walking speeds (km/hour) of the two groups of workers in two seasons

Groups	Parameters	*F* value	ANOVA significance
Brick carriers (*n*=22)	With load (summer) vs. with load (winter)	19.43	*p*<0.01
	Without load (summer) vs. without load (winter)	6.47	*p*<0.05
	With load (summer) vs. without load (summer)	10.04	*p*<0.01
	With load (winter) vs. without load (winter)	7.33	*p*<0.01
Brick molders (*n*=18)	With load (summer) vs. with load (winter)	5.12	*p*<0.05
	Without load (summer) vs. without load (winter)	7.25	*p*<0.01
	With load (summer) vs. without load (summer)	10.31	*p*<0.01
	With load (winter) vs. without load (winter)	5.01	*p*<0.05

*Load* means carrying 10 bricks (1 brick=~4.5 kg) at a time (carriers) and carrying mud (~40 kg) by pushing a small wooden cart (molders). However, most of the time, the molders work by sitting in a squatted posture, rather than by carrying mud (4).

## Discussions

### Questionnaire study

Most of the female workers are aware of their heat stress symptoms (about 72%), but still they do not have the knowledge of preventive measures to avoid them, nor do they take suitable precautions for them. As they come from lower socio-economic backgrounds, to earn more money they bear the heat stress (until conditions are extreme for them).

### Physical characteristics

It has been reported that the maximum working capacity of the industrial workers decreases with age after about 30 years of age (32). But here, most of the subjects were comparatively of younger age (younger than 30 years) and have much less work experience.

### Environmental parameters

According to the American Conference of Governmental Industrial Hygienists, WBGT should be 32.2°C for light work, 31.1°C for moderate work, and 30.0°C for heavy work, but these threshold exposure values (TLVs) were based on the assumption that the workers should be acclimatized, be fully clothed, and have adequate water and salt intake (33). None of the above said conditions were maintained in the brick-manufacturing units. Neither do they take rehydration solution in the hotter months, nor are they sufficiently acclimatized, as all of the workers are migrants. Thus, it is seen that (Fig. 1a and 1b) the most comfortable months for work are December and January, which have significantly lower temperatures (*p*<0.01) and lower relative humidity (*p*<0.05) than the other months of work. The most uncomfortable months are March, April, and May, which have significantly higher temperatures (*p*<0.01) and higher relative humidity (*p*<0.05) than the other months. So, among the 8 months of work, the female brick carriers are heat stressed for most of the time. Regarding their clothing, married female workers in both the winter and the summer seasons wear traditional Indian dress (i.e. the sari), and the younger or unmarried women wore skirts and shirts. As the fabric of these dress materials is not cotton but synthetic, this may also add to the heat stress of the female workers in the summer months. The current System of International Standards (ISO) not only relies on the thermal conditions experienced by the workers (ISO 7726) and the metabolic heat production due to work (ISO 8996) but also on the thermal properties of clothing (ISO 9920) (34). Thus, using personal protective devices (PPDs) such as sun-reflecting hats or working at dawn and at dusk with enough illumination would combat the extreme temperatures, especially in the summer months.

### Productivity analyses

Heat exposure and continued work cause fatigue and exhaustion, thus decreasing productivity (Fig. 2a and 2b). Work intensity must be slowed down to reduce internal body heat production, cardiac strain, and heat exhaustion (6). This ‘self-pacing’ for health protection reduces work productivity (9). The few studies that have quantified work productivity in relation to heat exposure in fieldwork situations did not document the cardiac strain to the same extent as this study has (35, 36). A study on rice harvesters in India showed that an increase of heat exposure (WBGT) of 1°C may cause a reduction of work productivity of approximately 5% (25). However, hourly productivity of the bricks molded or carried has not been monitored in this study. Thus, each degree rise in temperature causes about a 1.81% loss of productivity of these female workers.

However, productivity is hampered in extreme climates (i.e. both in summer and in winter seasons). Thus, only the optimum temperature is required for workers’ full efficient work and thus productivity. Moreover, raw clay bricks take more time to dry in the winter months, and it may take as long as 5 days for the complete drying that makes them fit for curing in the kiln. In contrast, bricks after molding completely dry in the open field in just 2 days. This may also cause the productivity to vary as well. But record books show that the wages of the female workers vary from 800 INR/week in the extreme summer months to 1,500 INR/week in the winter months. However, extreme winter seasons do not persist for long in the tropical countries, and thus altogether the productivity somewhat increases after the second week of November but gradually again decreases from the last week of December to January (colder months).

### Cardiovascular parameters

Studies carried out on workers in other industrial settings show that the effect of heat stress has been most significant on the cardiovascular system. It was observed that an easy job (a job that can be easily performed by the workers’ own capacity without any strain) became difficult at a temperature of 100°F (37.8°C) and at relative humidity over 85% (37). On the contrary, it is seen that air temperature above 34°C and heavy work affect the heart rate (Fig. 3) of the workers.

Many authors have used HR_rest_ as an indicator of physical workload related to muscular activities (38, 39). As the muscular activities are constant, so an increase in temperature in the summer months probably increases the HR_rest_ and thus the overall workload of these female workers. Moreover, women show more signs of tiredness and do not perceive fatigue the same way as men do (40). Even the low body weight of these workers may also affect their heart rates due to their poor nutrition and health. These data for this working class are also difficult to compare with the physiological models of Western countries.

Studies suggest that as continuous work in high environmental conditions gives rise to strain, so frequent breaks reduce the heat stress of the workers (14). Moreover, a good night's sleep is an important factor in trying to achieve maximum efficiency for working in a hot environment (15). The brick carriers are paid per thousand of bricks carried, called a *furan*. So to earn more, they carry more and more bricks until they are extremely fatigued. Again, they neither get sufficient rest after their work hours nor have a good night's sleep because they have to manage all of their household chores from cooking to rearing their children. Thus, altogether, they are at a further disadvantage compared to their male counterparts.

Occupational health risks and reductions of work productivity are linked to the combined influences of the energy expenditure required for a job and the workplace heat conditions (6, 9). Fatigue is experienced when the cardiovascular system cannot furnish sufficient oxygen to the muscles (29). Profuse sweating leading to daily severe dehydration can possibly also lead to chronic kidney disease (41, 42). Langkulsen et al. (43) found that hourly productivity among workers in Thailand was reduced in the construction and pottery industries, ranging from 10 to 60% depending on the level of heat exposure. Climate conditions in Thailand potentially affect both health and productivity in occupational settings. Besides, workers can also experience severe psychological distress caused by heat-related exhaustion (44). Moreover, increases in the local ambient temperature due to climate change may impact both workers’ health and economic conditions (45). Workers in low- and middle-income tropical countries are the worst affected (9). The female brickfield workers do have access to safe underground water for their cooking, drinking, and washing purposes. Wells and deep underground tube wells are generally the source of this water. Washing of clothes and utensils is also done in ponds or rivers situated nearby the brickfields. Unfortunately, sanitation is very poor, and the workers use open fields for this purpose.

The AWHR is much greater than its recommended values (46, 47) in winter months, and thus it is significantly much higher in the summers for both the brick carriers (*p*<0.01) and the brick molders (*p*<0.05). It can be further concluded (Table 3) that the work of the brick carriers is more stressful, as the HR_p_, NCC, and RCC of the brick molders are significantly less than those of the brick carriers working in both the summer and the winter seasons. However, heart rates are also influenced by one or more of a combination of factors, such as health conditions, aerobic capacity, age, weight, and thermal environment, and this change in the thermal environment probably causes the different resting heart rates in the ‘winter’ and the ‘summer’ months. Thus, rescheduling the work–rest cycle, along with the intake of fluid (rehydration solution is preferred) at regular intervals, especially in the summer months, would combat the extreme workload as well as the extreme temperature in the field.

### Analyses of the walking speed

As this is self-paced work, the female carriers reduced their speed of work. Thus, to reduce cardiac stress and fatigue, they increased their cycle time of carrying (3), and also their productivity decreased comparatively.

During continuous work in the heat, central nervous blood volume decreases as the cutaneous vessels dilate. The stroke volume falls and the heart rate increases to maintain the cardiac output. The effective circulatory volume also decreases as water is lost through sweating (48).

It is well reported that the maximum load carried at a comfortable speed without having much effect on an individual's health and efficiency is one-third of an individual's body weight for an 8-hour duration of work (49), but in this case the workers work beyond the 8-hour duration. In winter, the female carriers almost jovially run the walking distance (0.6±0.13 km) without the load, but in the summer seasons they get additionally stressed in the hot and humid environment (Table 4). Since speed is also related to these enhanced physiological responses, along with the weight carried by the workers, proper attention must be paid to this, or else it could lead to cumulative physiological changes in different systems of the workers’ health over time (29, 50). But these brick carriers cannot abstain from running as they have to carry more bricks or mud within a day's time to earn more and add more to the family income. Moreover, they are mostly unmarried and younger than the brick molders. So, the molders have to work faster to finish their task in the field and also complete their household duties. In addition, a study showed that the molders take 6.9±0.45 min to complete one cycle of work, and this amount of time does not significantly vary much between the two seasons (4).

There were some constraints in the field, so the heart rate was measured for only 30 min. Moreover, recording of hourly productivity along with the heart rate measurement and the hourly temperature measurement would actually add clarity to the picture in the brickfields of West Bengal. Still, this is now one of the limitations of the study, and a better understanding of the relationships between the productivity, the temperature, and the cardiac parameters would cause the authors to come to a definite conclusion.

## Conclusion

From analyses of the results of the cardiovascular parameters, it can be concluded that increased heat exposure increased the workload of the female brickfield workers. Due to continuous heat exposure, the workers slow down their working speed and increase their cycle time to cope with the additional stress of the workload, and this hampers their work productivity and consequently their income. There is a linear decline in productivity with an increase in maximum air temperature above 34.9°C, and the lost productivity for every degree rise in temperature is about 2%.

Thus, these female workers may also take up work as maid servants or as governesses, which may be an alternative but potential source of earning in their native places, and need not be migrants. Regarding policies in India for the unorganized sectors, there are the Factories Act, 1948, and the Building and Other Construction Workers (Regulation of Employment and Conditions of Service) Act, 1996, but none of these policies clearly explain the protection or rights of these female workers regarding their occupational health and safety.

Hence, ergonomic interventions, including rescheduling of the work–rest cycle, frequent fluid intake to replace the water lost due to sweating, using PPDs to protect themselves from radiating heat, and working at dawn or after sunset with sufficient lighting may help reduce the heat stress of the female brickfield workers. Moreover, this new evidence can also be used to estimate the future productivity losses and economic growth of the country.
